# Method for Detecting Non-Fat Milk Solids Content in Camel Milk Based on Dielectric Spectrum Technology

**DOI:** 10.3390/foods15203584

**Published:** 2026-10-09

**Authors:** Aliye Tuergong, Hong Zhang, Jingchi Guo, Yifei Gao, Shengkun Dong, Ting Zhao, Hong Zhang, Yang Liu

**Affiliations:** 1 Modern Agricultural Engineering Key Laboratory at Universities of Education Department of Xinjiang Uygur Autonomous Region, Tarim University, Alaer 843300, China; aliyetuergong@163.com (A.T.); 120230116@taru.edu.cn (H.Z.); 10757242273@stumail.taru.edu.cn (J.G.); 17648217086@163.com (Y.G.); dongshengkun2025@163.com (S.D.); tingzhao0809@163.com (T.Z.); 120050025@taru.edu.cn (H.Z.); 2 College of Mechanical and Electronic Engineering, Tarim University, Alaer 843300, China; 3 College of Hydraulic and Architectural Engineering, Tarim University, Alaer 843300, China

**Keywords:** solids-not-fat, dielectric spectroscopy, camel milk, machine learning, preprocessing methods

## Abstract

The difficulty in accurately detecting the nutritional components of camel milk severely restricts the development of the industry. Therefore, rapidly achieving non-destructive detection of the solids-not-fat content (SNFC) in camel milk is key to resolving this issue. This study was conducted on 400 camel milk samples collected from farms in five different regions of Xinjiang, China. Based on dielectric spectroscopy, this study proposes a quantitative detection method for camel milk SNFC that combines preprocessing with feature variable extraction. A network analyzer and an open-ended coaxial probe were used to collect the relative permittivity (ε′) and dielectric loss factor (ε″) of camel milk at 201 frequency points over the frequency range of 0.1–26.5 GHz. The raw dielectric spectra were preprocessed using standard normal variate (SNV), Savitzky–Golay smoothing (SGS), and Savitzky–Golay derivative (SGD) methods. Subsequently, feature variables were extracted from the preprocessed full dielectric spectra (FDS) using uninformative variable elimination (UVE), successive projections algorithm (SPA), and their combination (UVE-SPA). Then, partial least squares regression (PLSR), support vector machine (SVM), long short-term memory (LSTM), and Gaussian process regression (GPR) models were constructed to predict camel milk SNFC. Finally, the optimal model was externally validated. The results showed that the combination of preprocessing and feature variable extraction methods could improve the accuracy of the camel milk SNFC prediction models. Among all methods, SGD preprocessing combined with UVE-SPA feature variable extraction achieved the best overall performance, and the SGD-UVE-SPA-GPR model was identified as the optimal model for predicting camel milk SNFC. Compared with the model constructed from raw dielectric spectra, the model after preprocessing combined with feature extraction exhibited significant improvements in overall prediction accuracy. Specifically, the coefficient of determination of the prediction set $R_{P}^{2}$ increased from 0.9055 to 0.9685, representing a relative improvement of 6.96%; the root mean square error of prediction (RMSEP) decreased from 0.0899% to 0.0541%, corresponding to a reduction of 39.82%; and the residual predictive deviation of the prediction set (RPDP) increased from 3.2813 to 5.6780, representing an increase of 73.04%. These findings can provide a theoretical basis and technical support for the development of automated detection equipment for camel milk quality.

## 1. Introduction

Camel milk is rich in nutrients essential to the human body, including essential fatty acids, lactoferrin, whey protein and vitamin C. It exerts antibacterial, anti-inflammatory and antioxidant effects as well as functions to improve lipid and blood glucose metabolism [1]. Boasting extremely strong market competitiveness, camel milk has garnered widespread attention from consumers and relevant industry practitioners [2]. Solids-not-fat (SNF) is a collective term for all components in milk other than fat and water, and it represents one of the most important quality indicators of camel milk. Its content directly reflects the nutritional value, quality, and the consistency and mouthfeel of dairy products [3,4,5]. Therefore, real-time monitoring of camel milk SNFC during commercial processing holds significant practical value. Currently, SNFC determination mainly relies on the calculated method and the empirical formula method [6,7]. However, these methods are cumbersome, time-consuming, destructive to samples, and suffer from large errors and low accuracy. Therefore, there is a need for a simple, rapid, and non-destructive detection method for determining camel milk SNFC. Such a method would not only enable dairy enterprises to achieve online quality monitoring during fresh milk production but also provide a theoretical basis and practical reference for dairy product quality and safety control.

Near-infrared spectroscopy, mid-infrared spectroscopy, dielectric spectroscopy, and other techniques have already been applied for the rapid detection of various compositional parameters in dairy products [8,9,10]. However, spectroscopic techniques suffer from limitations in penetration depth and interference from fat globules or fat biofilms at boundary layers, which result in poor repeatability and suboptimal prediction accuracy [11]. In contrast, dielectric spectroscopy, owing to its high penetration capability and broadband frequency characteristics, overcomes the drawbacks of spectral analysis techniques associated with penetration depth limitations and boundary-layer fat globules or biofilms, thereby substantially improving prediction accuracy [12]. Dielectric spectroscopy, as a rapid and non-destructive method, has been widely applied in the field of dairy product detection. For instance, Liang et al. [13] employed dielectric spectroscopy combined with UVE, SPA, and UVE-SPA methods for feature variable extraction, and subsequently constructed multiple models, including PLSR, SVM, DT, RF, and LSBoost, to predict protein content. Fang et al. [14] utilized dielectric spectroscopy over the frequency range of 20–4500 MHz to extract relaxation parameters via a modified Debye model, and then combined VIP and SPA feature variable methods with SVR, PLSR, and LSSVM modeling to predict the protein content of bovine milk. Li et al. [15] employed dielectric spectroscopy in the 20–4500 MHz range to extract segmented dielectric relaxation parameters through Cole–Cole plot segmentation and a modified Debye model, and combined these with the SPA feature variable method and SVR modeling to predict the fat content of bovine milk. Yang et al. [16] employed dielectric spectroscopy to extract dielectric relaxation parameters via the Debye model and utilized models such as GRNN to predict the somatic cell count in bovine milk. The aforementioned studies indicate that raw dielectric spectral data contain a large amount of redundant information and noise signals that are not directly related to the target component, and direct modeling with such data can easily lead to model overfitting, poor stability, and decreased prediction accuracy. As a chemometrics method, feature variable extraction can eliminate uninformative and interfering variables, thereby reducing data dimensionality, removing multicollinearity among variables, and improving the prediction accuracy of models. Therefore, the combination of dielectric spectroscopy with feature variable extraction methods effectively improves the predictive performance for dairy product quality indicators [17,18]. Although the aforementioned studies have employed feature variable extraction methods to reduce data redundancy to a certain extent, dielectric spectroscopy measurements are susceptible to fluctuations in environmental temperature, instrument status, and other interfering factors. Consequently, raw dielectric spectra without preprocessing often contain noise signals, which impair the model’s ability to extract valid information and ultimately result in insufficient generalization ability and robustness, making it difficult to effectively eliminate the influence of spectral noise and baseline drift [19]. The preprocessing methods effectively remove noise and interference from the raw dielectric spectra. Feature variable extraction further screens the most strongly correlated variables with the target component from the preprocessed data, thereby reducing data dimensionality and eliminating multicollinearity [20,21]. Therefore, preprocessing is necessary to eliminate noise and baseline drift, followed by feature variable extraction to enhance prediction accuracy. Studies have shown that the combination of preprocessing and feature variable extraction can significantly improve the prediction accuracy and stability of models [22]. For instance, Guo et al. [23] employed dielectric spectroscopy with SNV spectral preprocessing, extracted feature variables using SPA, CARS, and a combination of CARS and SPA, and subsequently established PLSR, MLR, ELM, and LSSVM models to predict the moisture content of milk. Therefore, the combination of these two approaches represents an effective strategy for improving the prediction accuracy of quality indicators in dairy products. However, to date, no study has been reported on the detection of camel milk SNFC using models established via dielectric spectroscopy combined with preprocessing and feature variable extraction methods. Accordingly, the present study aims to enhance the prediction accuracy of dairy product quality indicators by integrating preprocessing with feature variable extraction.

This study aims to establish a rapid and non-destructive detection method for camel milk SNFC based on dielectric spectroscopy. The specific objectives are as follows: (1) to analyze the dielectric spectral characteristics of camel milk samples and perform descriptive statistical analysis on their SNFC data; (2) to compare the predictive performance of three spectral preprocessing methods across different models and determine the optimal preprocessing approach; (3) to screen characteristic frequency variables that are highly correlated with SNFC using UVE, SPA, and UVE-SPA feature variable extraction methods; (4) to construct four algorithms, namely PLSR, SVM, LSTM, and GPR, and establish quantitative prediction models for camel milk SNFC; and (5) to evaluate the predictive performance of the models under the optimal preprocessing and feature variable extraction conditions, identify the optimal prediction model, and perform external validation.

## 2. Materials and Methods

### 2.1. Experimental Materials and Sample Preparation

The experimental samples were obtained in November 2024 from five different farms in Xinjiang, China, namely: Keping County (Aksu Prefecture), the 7th Regiment of the First Division, Fuhai County (Altay Prefecture), Huocheng County (Ili Prefecture), and Yiwu County (Hami City). All samples were collected from fresh raw milk of healthy adult Bactrian camels at each farm. Forty samples were collected from each farm, yielding a total of 200 samples (40 replicates × 5 farms), with each sample volume being 30 mL. In April 2025, the same sampling protocol was implemented at the five farms, with 40 samples collected from each farm, yielding an additional 200 camel milk samples (as an independent external validation set), each with a volume of 30 mL. All collected samples were promptly placed in a low-temperature refrigerator at approximately 4 °C within 30 min and rapidly transported to the laboratory via cold-chain logistics. Camel milk sample collection was carried out under the natural production conditions of the Xinjiang region. All dielectric property measurements were uniformly conducted at the Key Laboratory of Modern Agricultural Engineering at Tarim University, where the ambient temperature was maintained at 20 ± 5 °C.

### 2.2. Measurement of Dielectric Parameters

**Figure 1:**
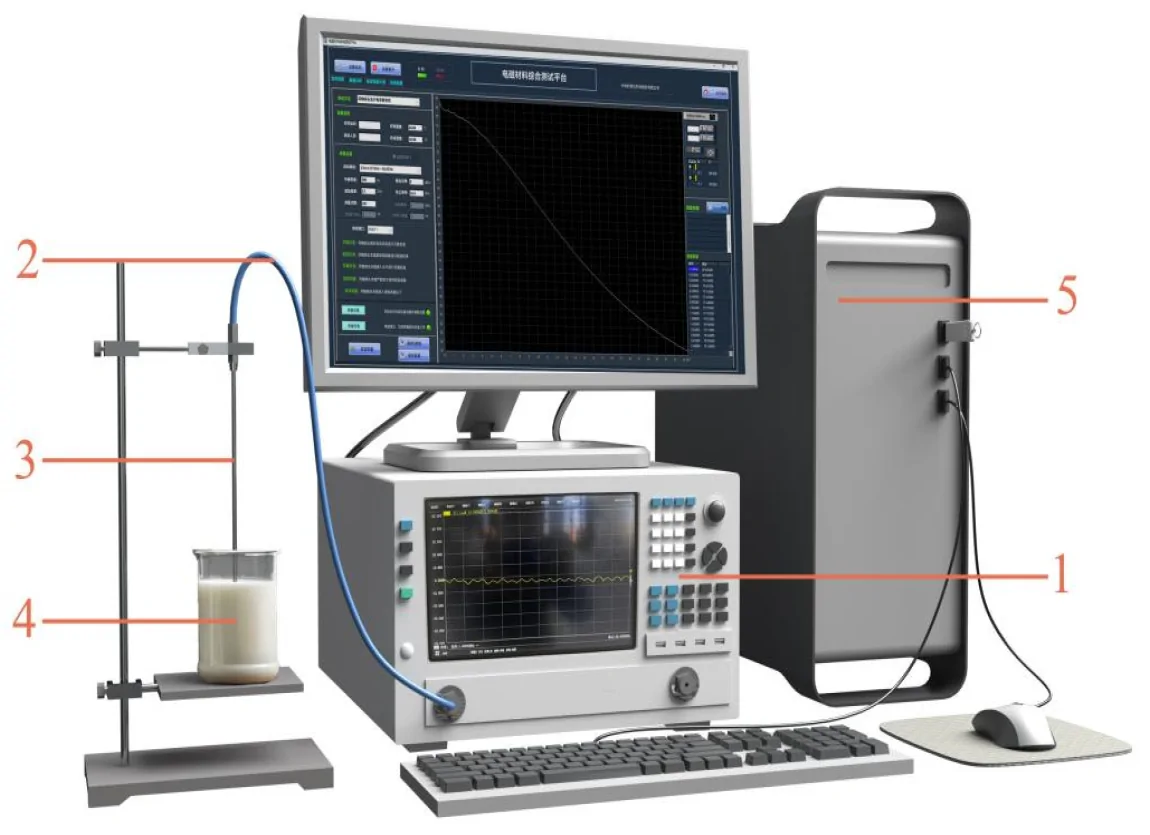
Camel milk dielectric parameter testing setup. 1. Vector network analyzer; 2. Connecting cables; 3. Open-ended coaxial probe; 4. Camel milk sample; 5. Computer.

Dielectric properties are inherent characteristics of materials and are commonly described by ε′ and ε″ [24]. As shown in Figure 1, a vector network analyzer (3671D, Ceyear Science and Technology Co., Ltd. of China Power Engineering Group, Qingdao, China) equipped with an open-ended coaxial probe was used to measure the ε′ and ε″ of camel milk over the frequency range of 0.1–26.5 GHz. Prior to measurement, the instrument was warmed up for more than 1 h, and the network analyzer was calibrated with the coaxial probe. The measurement parameters were set as follows: logarithmic scale, scanning frequency range of 0.1–26.5 GHz, and a total of 201 scanning frequency points. Accordingly, two dielectric spectra (ε′ spectrum and ε″ spectrum) were obtained for each sample, yielding a total of 402 data values (201 for ε′ and 201 for ε″). All feature variables used in subsequent modeling were derived from this set of frequency point response data of ε′ and ε″.

In this study, as the frequency increased from 0.1 GHz to 26.5 GHz, the 201 values of ε′ were sequentially numbered 1 to 201, and the 201 values of ε″ were numbered 202 to 402. Before instrument testing, a complete calibration procedure was performed. The calibration standards used included four types: open, short, 50 Ω matched load, and deionized water. First, vector network port calibration was performed by successively connecting the open, short, and standard 50 Ω matched load to complete the three-parameter port calibration, thereby eliminating measurement deviations introduced by the instrument’s internal circuitry. Following this, an open-short calibration was performed at the probe tip using air (open) and a standard short, to further eliminate systematic bias caused by the parasitic parameters of the probe itself. The complete calibration procedure was repeated before each batch of sample testing. In addition, the instrument baseline status was checked before and after each experiment. Once the baseline exhibited a noticeable shift, probe calibration was immediately re-performed to suppress measurement errors caused by long-term instrument drift. For measurement, each camel milk sample was thoroughly shaken and then poured into a beaker. The beaker was fixed on a lifting stand and uniformly adjusted to a preset height, so that the coaxial probe was immersed into the sample and kept stationary. Three consecutive replicate measurements were performed on the sample in the beaker, and the average value was taken as the final measured dielectric parameters of that sample. After the sample was loaded, the probe measurement position was changed and the measurement was repeated. Based on this procedure, the external validation samples were measured following the same protocol to evaluate the repeatability and reproducibility of the entire detection method. Immediately after the dielectric parameters of the quantitative samples were measured, the SNFC was determined according to the national standard method GB 5413.39—2010 [6].

### 2.3. Data Preprocessing

Appropriate preprocessing is beneficial for reducing noise and enhancing resolution [25,26]. In this study, SNV, SGS, and SGD methods were respectively applied to process the FDS. SNV effectively eliminates scattering effects and reduces multicollinearity in the data [27]. SGS preprocessing reduces noise, smooths the spectral data, and preserves the salient features of the signals [28]. SGD eliminates baseline drift and offset, thereby enhancing the data characteristics [29]. In this study, the sliding window size for SGS and SGD preprocessing was set to 7, and the polynomial order was set to 3. For SGD, a first-order derivative was used (derivative order = 1).

### 2.4. Methods for Extracting Feature Variables

Raw dielectric spectral data usually contain a substantial amount of redundant or repetitive information, which not only complicates model construction but also significantly slows down computation speed and may even impair the model’s discriminative ability [30]. Therefore, it is necessary to introduce optimal dielectric variable selection techniques to identify the core variables or characteristic features that can most effectively represent the original dielectric spectra. Both UVE and SPA are commonly used variable selection methods in chemometrics and data analysis, which can effectively reduce data dimensionality and extract informative features. UVE eliminates variables that contain less information than noise, thereby simplifying the model, avoiding overfitting, and improving model efficiency and accuracy [31]. In this study, UVE employed PLS regression with leave-one-out cross-validation. The number of latent variables was optimized within the range of 5–15, and the optimal number of PLS latent variables was determined within this range. SPA enhances modeling speed and prediction accuracy by reducing correlations among variables and selecting variables with minimal redundant information [32]. SPA performs forward variable search through orthogonal projection to eliminate multicollinearity. The maximum number of candidate variables needs to be manually set to a reasonable upper limit to control computational complexity. In this study, this upper limit was preset to 35, and the minimum number of candidate variables was fixed at 1. In this study, the optimal number of PLS latent variables for UVE and the optimal number of feature variables for SPA were both determined using the minimum RMSE of the calibration set as the selection criterion, following the method reported by Guo et al. [33]. The UVE-SPA method is a combination of UVE and SPA, in which UVE is first applied to select informative variables, and SPA is then used to select variables with the least redundant information from the informative variable set. Through this combined approach, the association between variables and the target attribute becomes more robust [34].

In this study, UVE, SPA, and UVE-SPA were employed for feature variable extraction to optimize model fitting and improve predictive performance.

### 2.5. Modeling Methods

Quantitative prediction models are mathematical tools that transform complex signals, such as dielectric spectra, into specific numerical values (e.g., content percentages), and they represent an important means of modern food safety detection and quality control [35]. In this study, PLSR, SVM, LSTM, and GPR models were established to predict camel milk SNFC, with ε′ + ε″ used as the input variables and SNF in camel milk as the output variable.

#### 2.5.1. Partial Least Squares Regression

PLSR is a supervised learning approach that combines the strengths of principal component analysis and multiple linear regression. By extracting latent variables, it characterizes the covariance structure between predictors and responses, enabling the construction of robust linear quantitative models. This method is especially well-suited for small-sample datasets characterized by high dimensionality and severe multicollinearity [36]. The PLSR model was implemented using MATLAB (R2024b)’ splsregress function. Prior to modeling, the input features and output true values were normalized to the [0, 1] interval using the mapminmax function to eliminate the effects of different feature scales. After training, denormalization was performed on the predicted results to restore the actual physical values. The dataset was randomly shuffled and divided into calibration and test sets at a ratio of 7:3. Specifically, 70% of the samples were used as the calibration (training) set for extracting principal components and training regression coefficients, while the remaining 30% served as the internal test set for evaluating model generalization performance. The core hyperparameter of the PLSR model is the number of latent variables. Since the information structure of the input datasets varies with different preprocessing methods and feature variable selection algorithms, the optimal number of latent variables is not uniform across all datasets. Therefore, the optimal number of latent variables was separately optimized for each input dataset, with candidate values ranging from 1 to 15. The optimal number of latent variables for each model was determined based on the comprehensive performance (*R*^2^, RMSE, and RPD) on the test set.

#### 2.5.2. Support Vector Machine

SVM is a supervised learning algorithm that constructs the optimal separating hyperplane by maximizing the class margin. Through the application of kernel functions, it transforms low-dimensional input data into a high-dimensional feature space, wherein the maximum-margin decision boundary is determined. This approach is particularly effective in managing overlapping data distributions [37]. In this study, the SVM model was adopted with a fixed second-order polynomial kernel, where the polynomial order was set to 2 and the kernel scale parameter was fixed at 2. The penalty parameter BoxConstraint and the ε-insensitive tube parameter Epsilon were not assigned fixed values manually; instead, they were adaptively calculated based on the interquartile range of the output labels in the training set. This strategy can adapt to different label magnitudes across datasets and reduce the subjective bias introduced by manual parameter tuning.

#### 2.5.3. Long Short-Term Memory

LSTM is a deep neural network architecture developed to overcome the short-term memory constraints inherent in conventional recurrent neural networks. By selectively memorizing informative features and discarding redundant or irrelevant information, LSTM effectively processes sequential data and mitigates the gradient explosion and vanishing gradient problems commonly encountered when recurrent neural networks are applied to long-sequence datasets [38,39,40,41]. In this study, model hyperparameters were optimized using a combination of grid search and manual tuning based on empirical experience. Specifically, the LSTM network was configured with one hidden layer, and the number of hidden units was set to 4, 16, and 32 for comparative evaluation. The optimal network structure was selected as: input layer → single-layer LSTM (4 units) → ReLU activation → fully connected output layer → regression layer, with ReLU adopted as the activation function of the output layer. The Adam optimizer was chosen for its suitability for small-sample regression tasks, fast convergence, and good robustness to hyperparameter selection. The initial learning rate was set to 0.01 with a piecewise decay strategy, where the decay factor was 0.1 and the decay step was set to 800–1200 epochs, meaning that the learning rate was reduced to 0.001 at the corresponding iteration rounds. Batch sizes of 16 and 32 were compared for selection, and the number of training epochs was set within the range of 1000–1500.

#### 2.5.4. Gaussian Process Regression

GPR is a non-parametric Bayesian modeling approach grounded in Gaussian process theory. It performs exceptionally well with small-sample datasets, offers robust convergence properties, and improves predictive accuracy, rendering it widely adopted across diverse domains such as machine learning and statistical analysis [42,43,44,45,46]. In this study, the GPR model was employed with the rational quadratic kernel as the covariance function, and the basis function was set to constant. The model hyperparameters were automatically optimized using maximum likelihood estimation. All hyperparameters were estimated on the training dataset via maximum likelihood estimation using MATLAB’s fitrgp function.

### 2.6. Model Evaluation

The performance of the models for predicting camel milk SNFC was evaluated using $R_{C}^{2}$, $R_{P}^{2}$, RMSEC, RMSEP, RPDC, and RPDP. $R_{C}^{2}$ reflects the goodness of fit of the regression model to the calibration set data, with values ranging from 0 to 1. The closer the value is to 1, the better the model fits the calibration data, indicating that the model can adequately explain the variability in the calibration set. $R_{P}^{2}$ reflects the predictive ability of the model on the prediction set data, also ranging from 0 to 1; the closer the value is to 1, the better the predictive performance of the model on the prediction set. RMSEC reflects the error level when predicting unknown samples during model training; a smaller value indicates higher predictive ability on the calibration set and a smaller deviation between predicted and actual values. RMSEP reflects the prediction error on the test set; a smaller value indicates stronger predictive ability for new data. RPD can be used to evaluate model accuracy: when RPD > 3, the model’s predictive performance is considered excellent; when 2.25 < RPD < 3, the performance is good; when 1.75 < RPD < 2.25, the model is basically acceptable; and when RPD < 1.75, the predictive performance is poor, indicating that the model is not practically applicable [47]. The calculations for $R_{C}^{2}$, $R_{P}^{2}$, RMSEC, RMSEP, RPDC, and RPDP are given by Equations (1)–(6), respectively:

(1) $$R_{C}^{2}=1-\frac{\sum_{i=1}^{n_{c}}(x_{i}-y_{i}{)}^{2}}{\sum_{i=1}^{n_{c}}(x_{i}-y_{c}{)}^{2}}$$

(2) $$R_{P}^{2}=1-\frac{\sum_{i=1}^{n_{p}}(x_{i}-y_{i}{)}^{2}}{\sum_{i=1}^{n_{p}}(x_{i}-y_{p}{)}^{2}}$$

(3) $$\mathrm{RMSEC}=\sqrt{\frac{1}{n_{c}}\sum_{i=1}^{n_{c}}(x_{i}-y_{i}{)}^{2}}$$

(4) $$\mathrm{RMSEP}=\sqrt{\frac{1}{n_{p}}\sum_{i=1}^{n_{p}}(x_{i}-y_{i}{)}^{2}}$$

(5) $$\mathrm{RPDC}=\frac{\mathrm{SD}}{\mathrm{RMSEC}}$$

(6) $$\mathrm{RPDP}=\frac{\mathrm{SD}}{\mathrm{RMSEP}}$$

where $x_{i}$ is the predicted value of SNFC for the i-th camel milk sample, $y_{i}\;$ is the measured value of SNFC for the i-th camel milk sample, $y_{c}$ and $y_{p}$ are the mean values of the calibration set and prediction set, respectively, $n_{c}$ is the number of camel milk samples in the calibration set, $n_{p}$ is the number of camel milk samples in the prediction set, and SD represents the standard deviation of all samples in the validation set for a given target parameter.

### 2.7. Model Development Workflow

**Figure 2:**
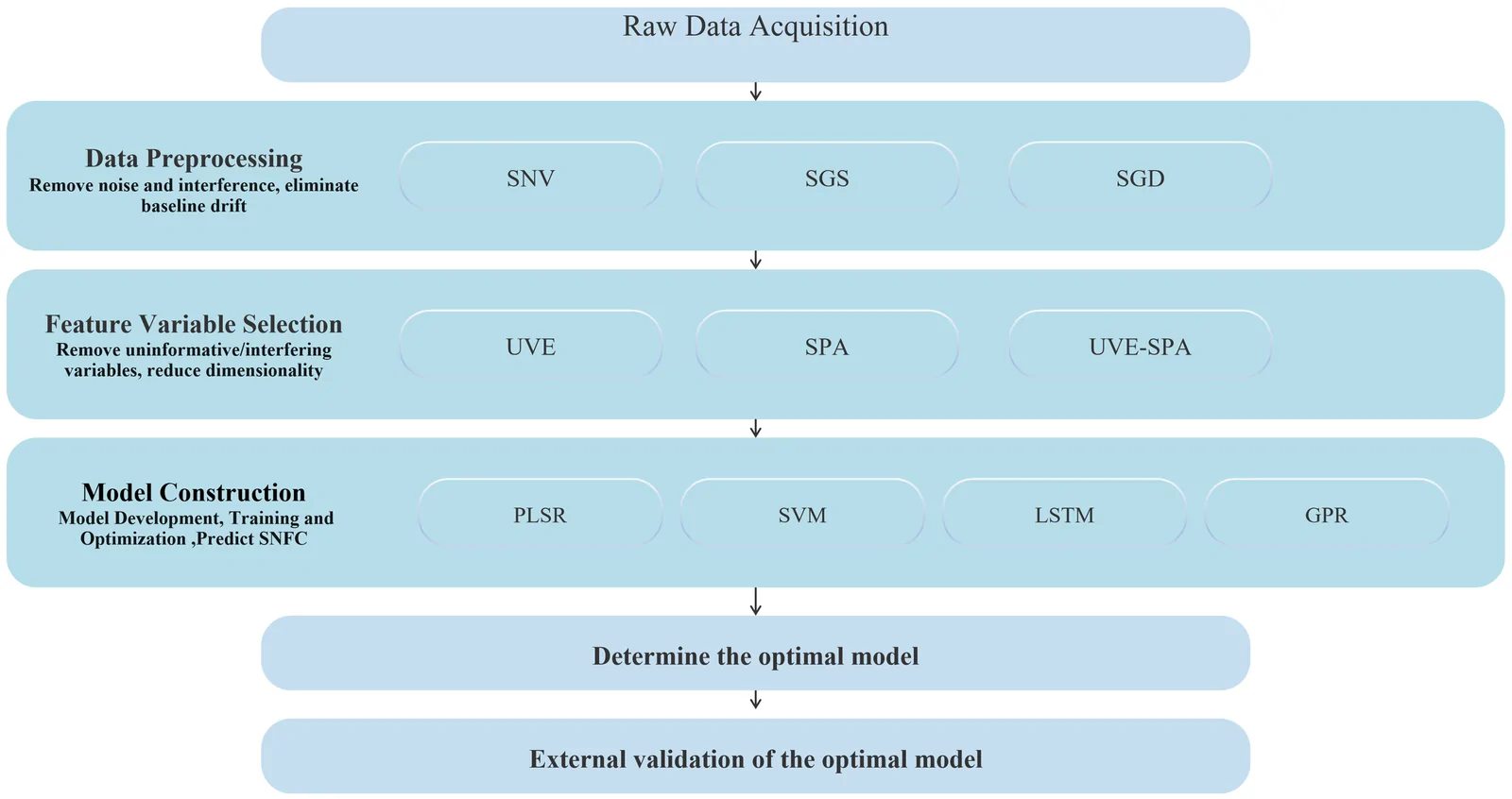
Model development and validation workflow.

This study established a model development and validation workflow, as shown in Figure 2. The overall workflow covers raw data acquisition, data preprocessing, feature selection, model development, training and optimization, determination of the optimal model, and external validation.

In the data preprocessing stage, three methods, namely SNV, SGS, and SGD, were employed. In the feature selection stage, three methods, namely UVE, SPA, and UVE-SPA, were compared. In the model development stage, four algorithms, namely PLSR, SVM, LSTM, and GPR, were used for modeling. For each combination, the dataset was divided into a calibration set and a prediction set, and optimal parameter tuning was performed. Through comprehensive evaluation metrics and model selection, the optimal model for this study was finally determined and externally validated using independent samples to evaluate its robustness and generalization ability.

## 3. Results and Analysis

### 3.1. Statistical Analysis of Camel Milk SNFC

As shown in Table 1, the statistical analysis of camel milk SNFC from different regions is presented. In terms of content range, the maximum SNF content in camel milk was 12.20%, and the minimum was 11.10%. At the overall level, the mean SNF content ± standard deviation was 11.51% ± 0.29%, with a standard deviation of only 0.29%, indicating that the SNFC differences among samples from different regions were small and the stability was good. These results can provide data support for subsequent quantitative modeling of dielectric spectra with SNF and feature variable extraction.

#### Distribution Characteristics of Camel Milk SNFC Data from Different Regions

**Figure 3:**
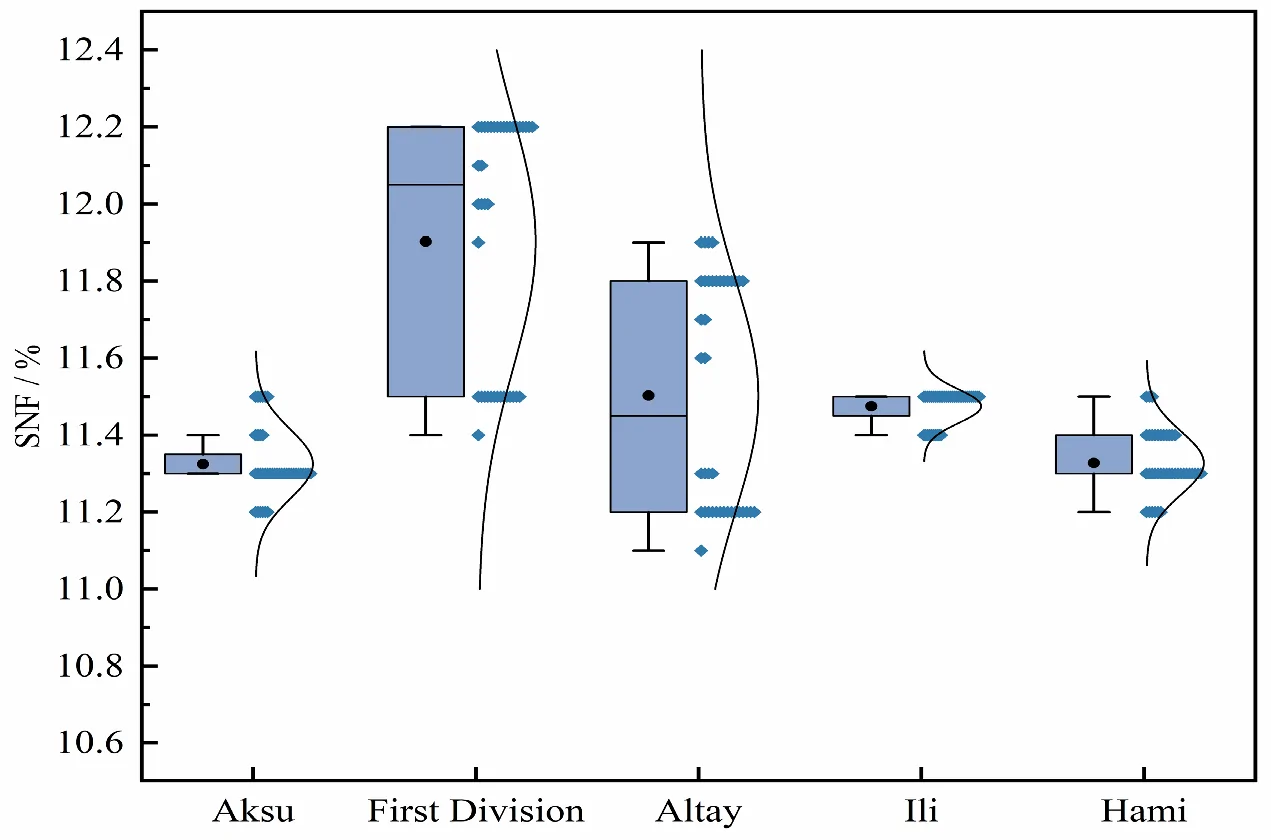
Distribution characteristics of camel milk SNFC data from different regions.

The distribution of camel milk SNFC data from different regions is shown in Figure 3. In the figure, the boxplots display the quartiles and dispersion of SNFC for samples from each region, while the outer density contour curves reflect the distribution patterns of the data in each group. It can be clearly observed that samples from the 7th Regiment of the First Division exhibited the highest overall SNFC values with relatively greater dispersion. Samples from Huocheng County (Ili Prefecture), Keping County (Aksu Prefecture), and Yiwu County (Hami City) showed more concentrated data with low dispersion. Samples from Fuhai County (Altay Prefecture) displayed intermediate SNFC levels. Combined with the distribution contours, the overall data trends for each group can be preliminarily assessed, providing a visual reference for evaluating the prerequisites for subsequent analysis of variance (ANOVA).

### 3.2. Frequency Dependence of Camel Milk Dielectric Spectra

**Figure 4:**
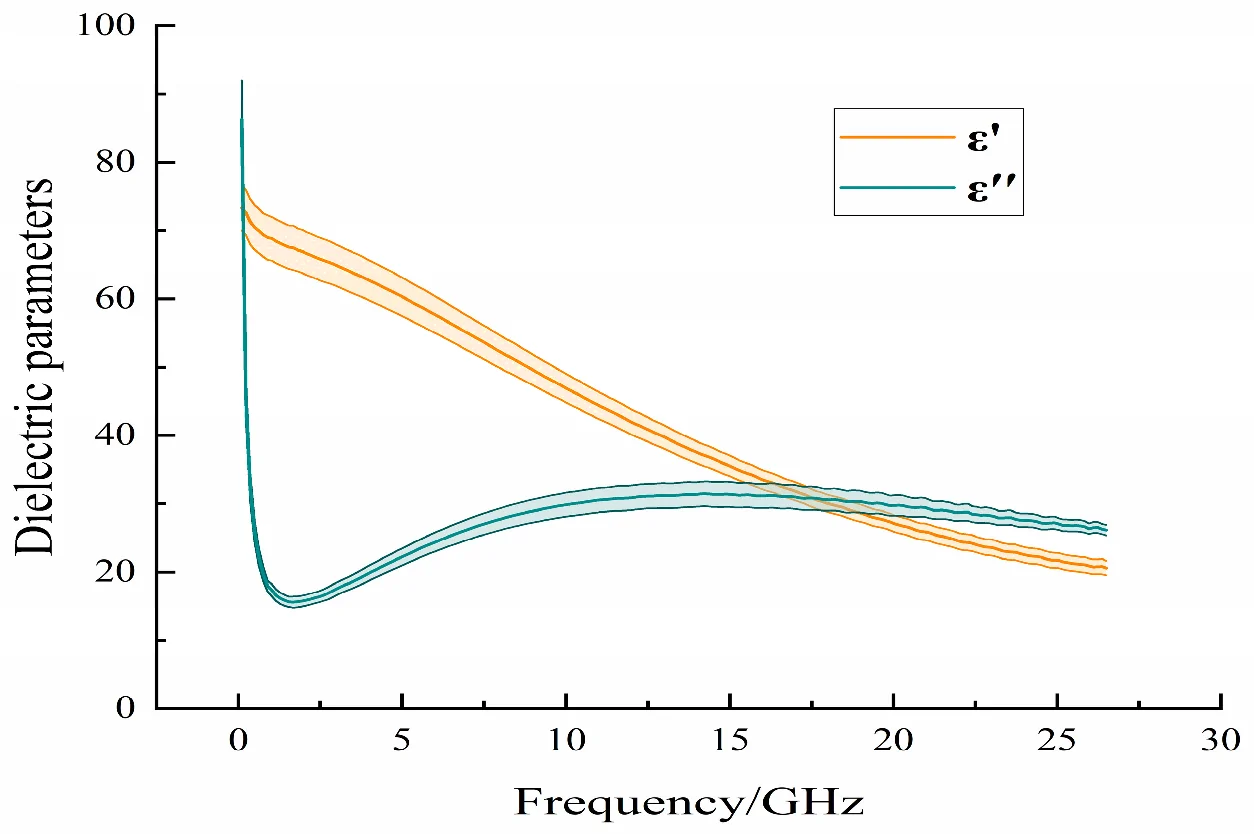
Frequency dependence of dielectric parameters ε′ and ε″ (mean ± standard deviation).

Figure 4 shows the mean and standard deviation of the dielectric spectra (ε′ and ε″) for all camel milk samples over the frequency range of 0.1–26.5 GHz. Overall, the ε′ of camel milk exhibits a continuous decreasing trend with increasing frequency, while ε″ displays a pattern of initially decreasing and then increasing. This frequency-dependent behavior is consistent with the microwave dielectric response characteristics of bovine milk [48]. ε′ represents the electrical energy storage capacity of the system, and its variation is primarily attributed to the dipole relaxation effect of polar molecules within the system. Under low-frequency alternating electric field conditions, polar dipole molecules—such as water molecules and proteins—can rapidly orient and rotate in response to the external electric field direction, resulting in full polarization, and thus ε′ remains at a relatively high level. As the test frequency increases, the alternating rate of the electric field direction accelerates continuously, and the rotational relaxation process of molecular dipoles cannot keep pace with the rapid field changes, leading to polarization lag. Consequently, the dipole orientation polarization is gradually weakened, ultimately causing ε′ to decrease progressively with increasing frequency [49]. ε″ reflects the degree of energy loss associated with the conversion of electromagnetic energy into thermal energy, and the loss primarily comprises two contributions: ionic conductivity loss and dipole relaxation loss. In the low-frequency range, the abundant free mineral salt ions in the camel milk system serve as the dominant source of loss, and the ionic conductivity loss exhibits a negative correlation with frequency, diminishing as frequency increases, which results in a decreasing trend in ε″. As the frequency further increases into the high-frequency microwave region, the dipole relaxation loss of water molecules becomes dominant. The high-frequency alternating electric field drives polar molecules to rotate rapidly back and forth, intensifying friction and collisions among molecules and between molecules and ions. The resulting energy loss from dipole relaxation increases rapidly, counteracting the decreasing effect of ionic conductivity loss, and ultimately causing ε″ to increase with further frequency elevation [50,51]. Thus, the low-frequency signals primarily reflect the conductive behavior of ionic components in camel milk, while the high-frequency region more prominently reflects the dipole relaxation responses of water molecules and macromolecules such as proteins.

### 3.3. Modeling Results

To achieve accurate prediction of SNFC in camel milk, a total of 200 camel milk samples were randomly partitioned into a calibration set and a prediction set in a 7:3 ratio. The calibration set comprised 140 samples and was employed for model construction, whereas the prediction set consisted of 60 samples and was utilized to assess the generalization ability of the established models. The feature variables derived from the preprocessed full dielectric spectra (ε′ + ε″) served as input predictors, with camel milk SNFC designated as the target output. On this basis, PLSR, SVM, LSTM, and GPR models were subsequently developed.

#### 3.3.1. Prediction of Camel Milk SNFC Based on Feature Variable Extraction Methods

Based on the raw dielectric spectral data (without preprocessing), four quantitative prediction models, namely PLSR, SVM, LSTM, and GPR, were established. The model performance was comprehensively evaluated using metrics such as $R_{C}^{2}$, $R_{P}^{2}$, RMSEC, RMSEP, RPDC and RPDP, as shown in Table 2. For the FDS data, the established PLSR, SVM, LSTM, and GPR models achieved $R_{P}^{2}$ values of 0.9017, 0.8902, 0.9091, and 0.9055, RMSEP values of 0.0920%, 0.1006%, 0.0886%, and 0.0899%, and RPDP values of 3.2168, 3.0434, 3.3259, and 3.2813, respectively. Compared with FDS modeling, the use of feature variable extraction methods effectively improved the prediction accuracy of all models. Among them, the PLSR model established with feature variables extracted via the UVE-SPA method exhibited the best performance, with $R_{P}^{2}$ = 0.9291, RMSEP = 0.0781%, and RPDP = 3.7880.

#### 3.3.2. Camel Milk SNFC Prediction Using Preprocessing Integrated with Feature Variable Extraction

While feature variable extraction alone is capable of excluding variables with negligible or random contributions to the model output and mitigating multicollinearity, the raw dielectric spectra still suffer from instrumental drift, ambient noise, and baseline shifts in the absence of preprocessing. To address these disturbances, the present study integrated preprocessing with feature variable extraction strategies for SNFC prediction in camel milk, with the objective of further improving both the predictive accuracy and model robustness.

##### Prediction of Camel Milk SNFC Based on SNV Combined with Feature Variable Extraction

On the basis of SNV preprocessing, PLSR, SVM, LSTM, and GPR prediction models were established using the raw dielectric spectral data, and feature variable extraction methods were further employed to optimize model performance. The prediction results are presented in Table 3. Compared with the modeling results without preprocessing (where $R_{P}^{2}$ values under FDS were all below 0.91), SNV preprocessing significantly improved the prediction accuracy of all models. Among them, all models established with the UVE-SPA feature variable extraction method exhibited good predictive performance: the PLSR model achieved an $R_{P}^{2}$ of 0.9354, an RMSEP of 0.0726%, and an RPDP of 3.9675; the SVM model achieved an $R_{P}^{2}$ of 0.9527, an RMSEP of 0.0638%, and an RPDP of 4.6413; the LSTM model achieved an $R_{P}^{2}$ of 0.9345, an RMSEP of 0.0752%, and an RPDP of 3.9187; and the GPR model achieved an $R_{P}^{2}$ of 0.9447, an RMSEP of 0.0705%, and an RPDP of 4.2894. It can be seen that the combined application of SNV and UVE-SPA feature variable extraction methods can effectively improve the detection accuracy of camel milk SNFC. The SNV-UVE-SPA-SVM combination exhibited the best predictive performance, with $R_{P}^{2}$ = 0.9527, RPDP = 4.6413, and RMSEP = 0.0638%.

##### SNFC Prediction in Camel Milk Using SGS Combined with Feature Variable Extraction

Following SGS preprocessing of the full dielectric spectra, four quantitative prediction models—PLSR, SVM, LSTM, and GPR were developed in conjunction with various feature variable extraction strategies for the determination of camel milk SNFC. The resulting predictive outcomes are summarized in Table 4. In comparison with the unprocessed FDS-based models, all models exhibited enhanced prediction accuracy subsequent to SGS preprocessing. Furthermore, the models constructed with SGS preprocessing combined with feature variable extraction generally outperformed their FDS counterparts. Under the SGS-UVE-SPA framework, the PLSR, SVM, LSTM, and GPR models all demonstrated high predictive accuracy and robustness. Notably, the SGS-UVE-SPA-GPR model delivered the best overall performance, attaining an $R_{P}^{2}$ of 0.9393, an RMSEP of 0.0755%, and an RPDP of 4.0949.

##### Prediction of Camel Milk SNFC Based on SGD Combined with Feature Variable Extraction

Based on SGD preprocessing, different feature variable extraction methods were further employed to reduce the dimensionality of the FDS, and four prediction models, namely PLSR, SVM, LSTM, and GPR, were constructed for quantitative analysis of camel milk SNFC. The results are presented in Table 5. After SGD preprocessing combined with SPA, UVE, and UVE-SPA for feature variable extraction, all established models achieved $R_{P}^{2}$ values higher than 0.93, RPDP values higher than 3.87, and RMSEP values lower than 0.0784%, demonstrating good predictive performance. Among them, the four models established with UVE-SPA feature extraction all achieved the best predictive performance: the PLSR model achieved an $R_{P}^{2}$ of 0.9410, an RPDP of 4.1511, and an RMSEP of 0.0750%; the SVM model achieved an $R_{P}^{2}$ of 0.9598, an RPDP of 5.0306, and an RMSEP of 0.0627%; the LSTM model achieved an $R_{P}^{2}$ of 0.9585, an RPDP of 4.9246, and an RMSEP of 0.0598%; and the GPR model achieved an $R_{P}^{2}$ of 0.9685, an RPDP of 5.6780, and an RMSEP of 0.0541%. The SGD-UVE-SPA-GPR combination delivered the optimal performance for predicting SNFC in camel milk, with $R_{P}^{2}$ = 0.9685, RMSEP = 0.0541%, RPDP = 5.6780.

#### 3.3.3. Feature Variable Selection Results Under Different Preprocessing Methods

**Figure 5:**
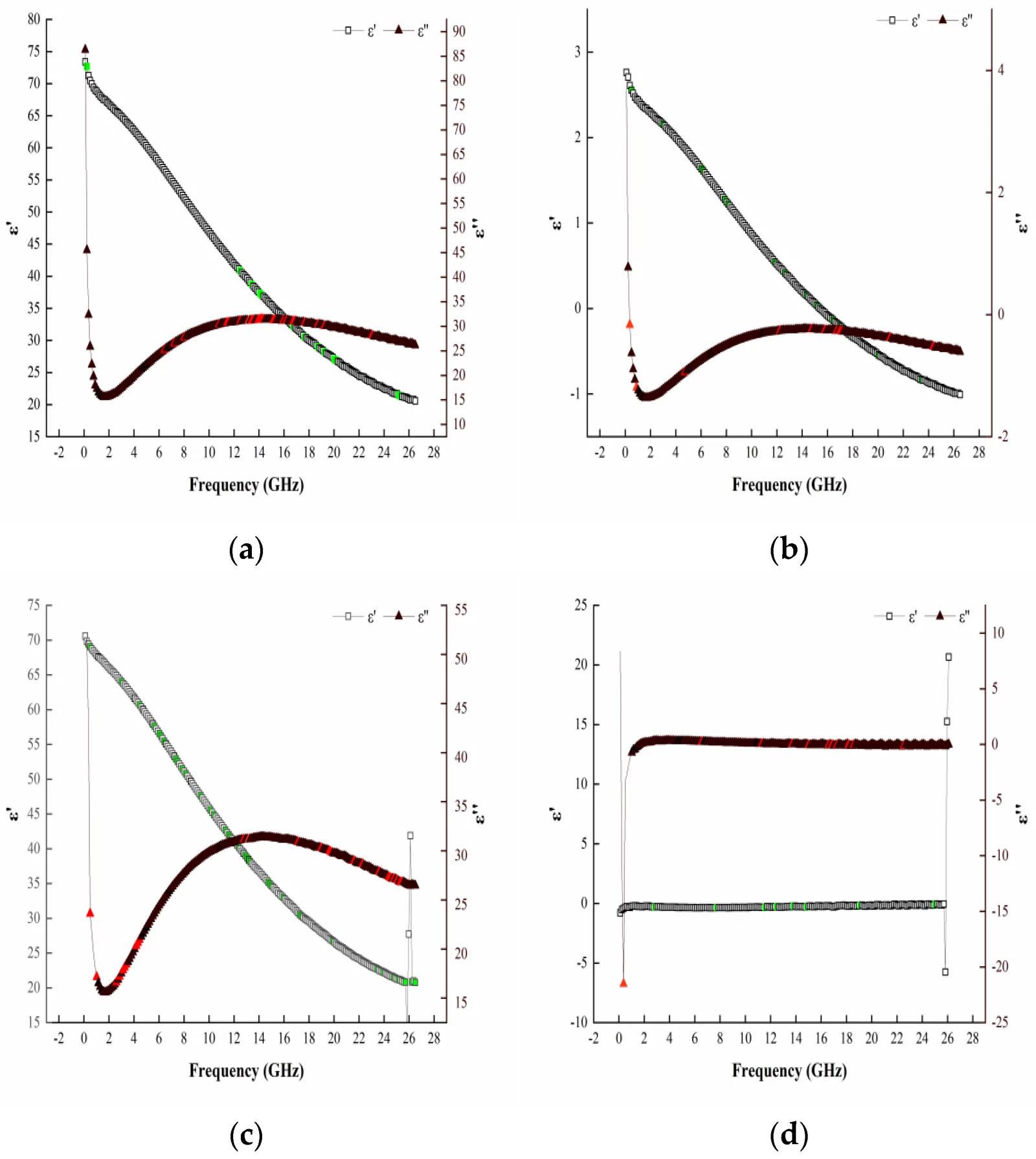
Results of feature variable selection for the optimal camel milk SNFC prediction models under various preprocessing approaches: (**a**) UVE-SPA-PLSR, (**b**) SNV-UVE-SPA-SVM, (**c**) SGS-UVE-SPA-GPR, and (**d**) SGD-UVE-SPA-GPR.

Figure 5 shows the feature variables selected from camel milk samples under different preprocessing methods. In the figure, the curve represents the full dielectric spectrum of a representative sample, and the red and green markers indicate the feature variables extracted by the feature variable extraction methods. As shown in Figure 5a, 30 feature variables were extracted using UVE-SPA of which 13 were from ε′ at frequencies of 0.232, 12.508, 13.432, 14.092, 14.224, 16.732, 17.656, 18.712, 19.240, 20.032, 20.164, 25.180, and 25.312 GHz, and 17 were from ε″ at frequencies of 6.436, 7.228, 8.020, 10.792, 11.452, 11.584, 13.432, 13.564, 13.960, 14.356, 15.148, 15.280, 15.940, 16.204, 16.732, 16.864, and 23.068 GHz. Out of the 402 feature variables in the FDS, the number of extracted feature variables accounted for only 7.46%. As shown in Figure 5b, after SNV preprocessing followed by UVE-SPA extraction, 25 feature variables were selected, of which 15 were from ε′ at frequencies of 0.496, 3.004, 6.040, 6.172, 7.888, 8.152, 11.848, 12.640, 14.356, 15.148, 16.468, 16.996, 17.260, 20.032, and 23.464 GHz, and 10 were from ε″ at frequencies of 0.892, 4.720, 12.244, 14.752, 15.808, 16.732, 16.996, 17.128, 20.955, and 24.388 GHz. Among the 402 variables in the FDS, the extracted feature variables constituted only 6.22%. As shown in Figure 5c, after SGS preprocessing followed by UVE-SPA extraction, 60 feature variables were selected, of which 38 were from ε′ at frequencies of 0.496, 3.004, 4.456, 5.644, 6.040, 6.172, 6.436, 7.360, 7.492, and 7.888 GHz, and 22 were from ε″ at frequencies of 1.024, 2.608, 2.872, 3.136, 3.268, 4.192, 4.324, 4.456, 12.772, 17.128, 18.976, 19.636, 19.768, 21.615, 22.275, 23.596, 24.388, 24.520, 24.784, 25.048, 25.180, and 25.440 GHz. Among the 396 variables in the FDS, the extracted feature variables accounted for 15.15%. As shown in Figure 5d, after SGD preprocessing followed by UVE-SPA extraction, 20 feature variables were selected, of which 9 were from ε′ at frequencies of 3.004, 7.888, 11.716, 12.640, 13.960, 14.356, 15.016, 19.240, and 25.180 GHz, and 11 were from ε″ at frequencies of 0.496, 6.700, 11.452, 13.828, 15.148, 16.732, 16.996, 17.392, 18.316, 18.712, and 22.672 GHz. Among the 396 variables in the FDS, the extracted feature variables accounted for only 5.05%. For the remaining feature variable frequency selections, please see the Supplementary File “Table of Model-Selected Frequencies”.

#### 3.3.4. Effects of Preprocessing and Feature Extraction on Model Predictive Performance

Without preprocessing, although the $R_{P}^{2}$ values of the models after feature extraction using UVE, SPA, and UVE-SPA were all superior to the direct FDS modeling results, there remained room for further improvement. Even though the UVE-SPA combination achieved the best performance among the non-preprocessed approaches (UVE-SPA-PLSR: $R_{P}^{2}$ = 0.9291, RMSEP = 0.0781%, RPDP = 3.7880), its overall prediction accuracy was still significantly lower than that of the SNV-UVE-SPA-SVM, SGS-UVE-SPA-GPR, and SGD-UVE-SPA-GPR models. This indicates that although relying solely on feature variable extraction can effectively reduce data redundancy and the number of variables in the dielectric spectra, common issues such as noise interference and baseline drift still persist in the spectral data. Therefore, preprocessing is a necessary prerequisite for enhancing model predictive performance.

Regardless of whether in PLSR, SVM, LSTM, or GPR models, the $R_{P}^{2}$ values obtained after SGD preprocessing were the highest, indicating that SGD can effectively eliminate spectral noise and baseline drift, and extract informative features strongly correlated with SNFC. In contrast, the overall performance metrics under SNV and SGS preprocessing were consistently lower than those under SGD.

Following SGD preprocessing, the further introduction of UVE, SPA, and UVE-SPA for feature variable extraction led to significantly better predictive performance for all models compared with those without feature extraction. This indicates that the combination of SGD preprocessing and feature extraction methods is superior to either approach applied individually, as it can effectively eliminate redundant variables from the FDS data while enhancing the representativeness of the retained feature information. Among them, the SGD-UVE-SPA combined method achieved the most prominent results, with $R_{P}^{2}$ values exceeding 0.95 across all models: SGD-UVE-SPA-PLSR = 0.9502, SGD-UVE-SPA-SVM = 0.9598, SGD-UVE-SPA-LSTM = 0.9585, and SGD-UVE-SPA-GPR = 0.9685.

### 3.4. Determination of the Optimal Model

**Figure 6:**
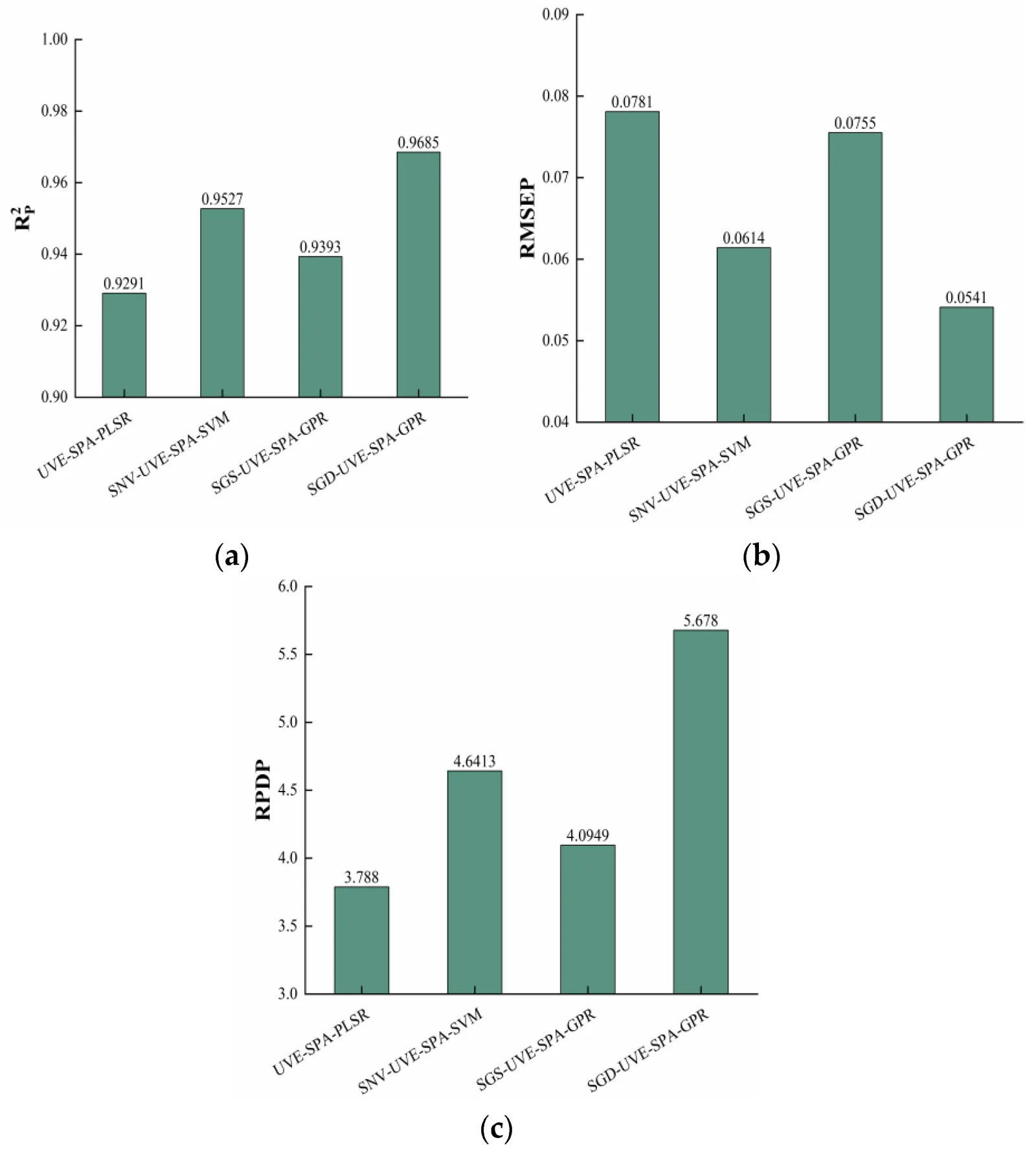
Selection of optimal models for camel milk SNFC under different preprocessing strategies ((**a**): $R_{P}^{2}$, (**b**): RMSEP, (**c**): RPDP).

To determine the optimal dielectric spectral prediction model for camel milk SNFC, the prediction accuracies of the best models under different preprocessing schemes were compared in this study. As shown in Figure 6, the optimal results for camel milk SNFC exhibited clear gradient differences. Among all models, PLSR demonstrated the worst overall prediction performance, with the lowest $R_{P}^{2}$ and RPDP and the highest RMSEP. In contrast, SGD-UVE-SPA-GPR achieved the optimal performance across all three evaluation metrics: it attained the highest $R_{P}^{2}$, indicating the best model fitting degree; the lowest RMSEP, reflecting the smallest prediction error; and the highest RPDP, demonstrating excellent predictive discrimination ability and stability. The SGD-UVE-SPA-GPR combined model significantly outperformed the other models in both prediction accuracy and model stability, and was therefore identified as the optimal prediction model in this study.

#### 3.4.1. Residual Analysis of the Optimal Model

**Figure 7:**
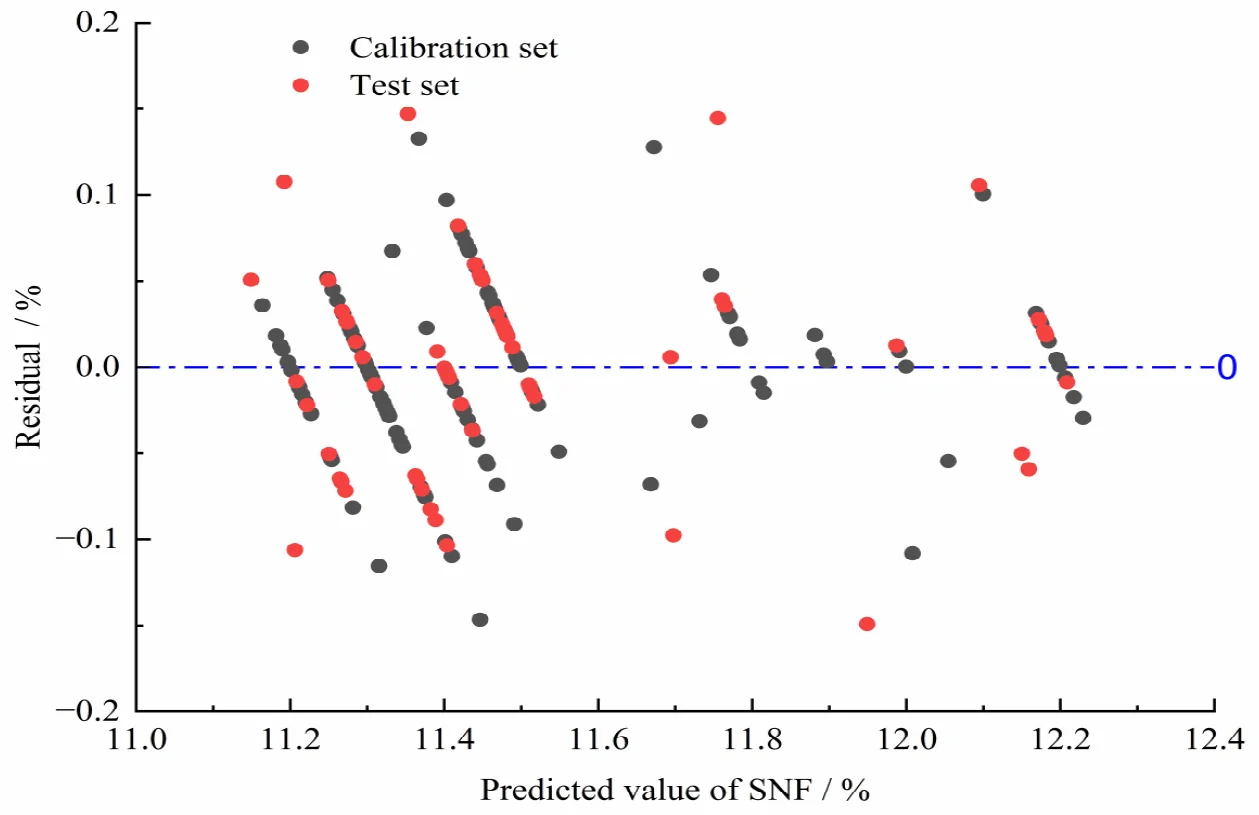
Residual-versus-predicted-value distribution for the SGD-UVE-SPA-GPR model.

To assess the goodness-of-fit of the SGD-UVE-SPA-GPR model, a residual versus predicted value scatter plot was generated, with the results presented in Figure 7. In this figure, black dots denote calibration set samples, red dots denote test set samples, and the blue dashed line represents the zero-residual reference line. Inspection of the residual distribution reveals that the residuals for both the calibration and test sets are randomly distributed on both sides of the zero-residual line, without any systematic tendency to cluster above or below the line, suggesting the absence of significant systematic bias. Furthermore, the residual spread does not widen with increasing predicted SNF values, and no evidence of heteroscedasticity is apparent. The similarity in residual patterns between the calibration and test sets provides preliminary evidence that the SGD-UVE-SPA-GPR model exhibits a robust overall fit.

#### 3.4.2. SHAP Interpretability Analysis of the Optimal Model

**Figure 8:**
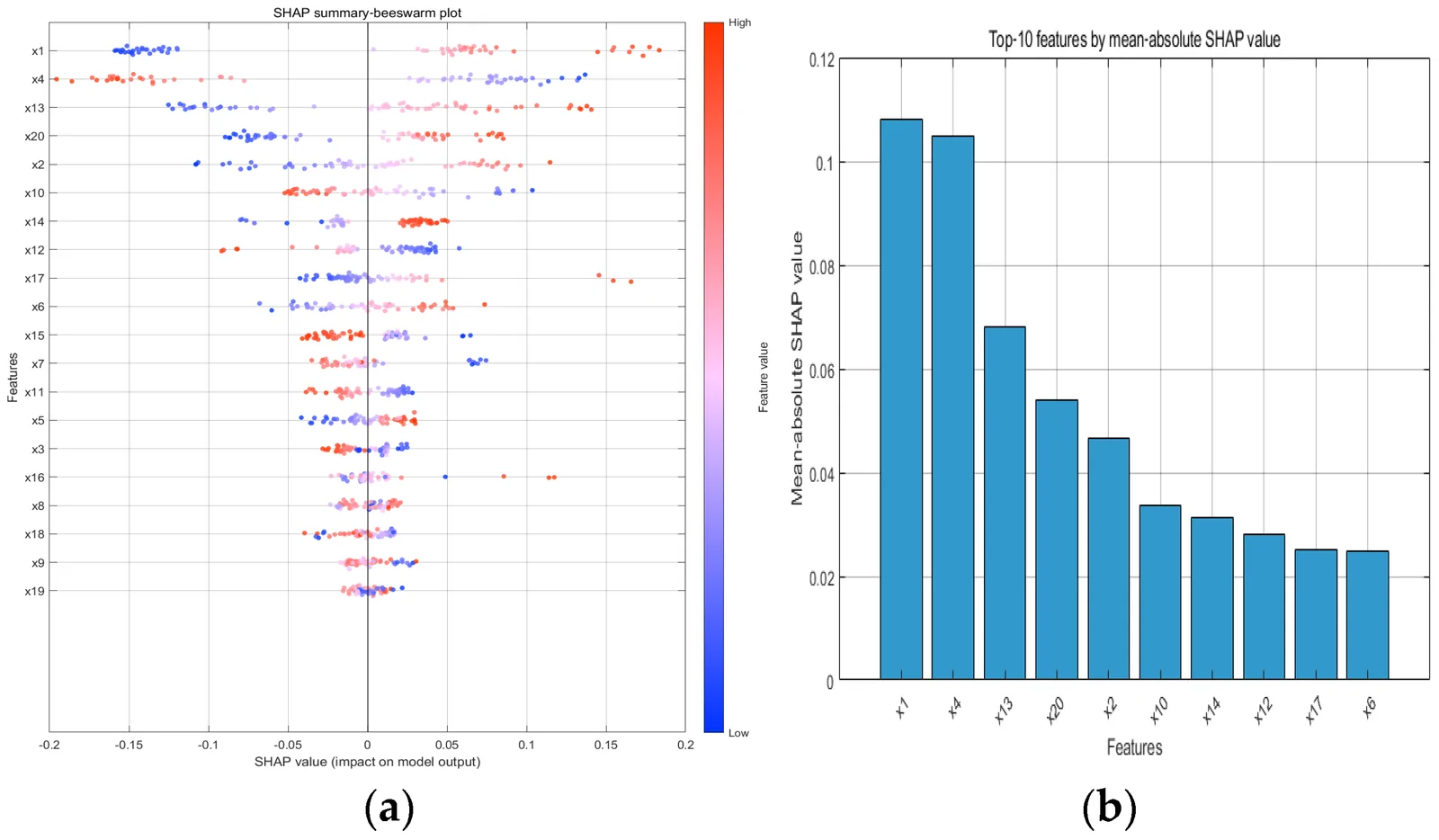
SHAP interpretability analysis results for the SGD-UVE-SPA-GPR model: (**a**) SHAP summary bee swarm plot; (**b**) bar chart of the top 10 features by mean absolute Shapley value.

To elucidate the prediction mechanism of the SGD-UVE-SPA-GPR optimal model and to quantify the contribution of each dielectric spectral feature to the prediction of camel milk SNF, SHAP (Shapley additive explanations) interpretability analysis was performed on the 20 input features across 60 samples in the test set, with the results presented in Figure 8. Figure 8a shows the SHAP summary bee swarm plot, where the horizontal axis represents the SHAP contribution value of each feature to the model’s predicted output, the vertical axis lists the input features, and the color of each point indicates the magnitude of the feature value itself. Figure 8b presents a bar chart of the top 10 features with the highest mean absolute Shapley values, quantitatively displaying the contribution strength of each feature to the model output. The SHAP analysis results reveal that different dielectric spectral features exhibit markedly distinct contributions to the prediction of camel milk SNF.Feature x_1_ exhibited the highest mean absolute Shapley value of 0.1081, establishing it as the primary driver of the model’s predictions. This was followed by x_4_ (0.1049), x_13_ (0.06816), x_20_ (0.05385), and x_2_ (0.04663), with these five features contributing significantly more than the remaining variables. The mean absolute Shapley values of x_10_, x_14_, x_12_, x_17_, and x_6_ decreased successively, categorizing them as secondary contributing features. As observed from the bee swarm plot distribution, higher values of certain features yielded positive SHAP contributions, driving the model toward higher predicted SNF values, while other features exerted negative contributions, reflecting the complex response relationships between different dielectric features and the target physicochemical indicator. These results indicate that the feature subset selected by SGD-UVE-SPA is not of uniform importance, with a small number of dielectric spectral features playing a dominant role in model prediction. The optimal model achieved excellent predictive performance, with $R_{P}^{2}$ = 0.9685 and RPDP = 5.6780. Furthermore, the SHAP interpretability analysis enabled the elucidation of the GPR black-box model by identifying the key input variables, thereby preventing the model from relying solely on statistical correlations and further reinforcing its reliability.

### 3.5. Validation of the Optimal Model

**Figure 9:**
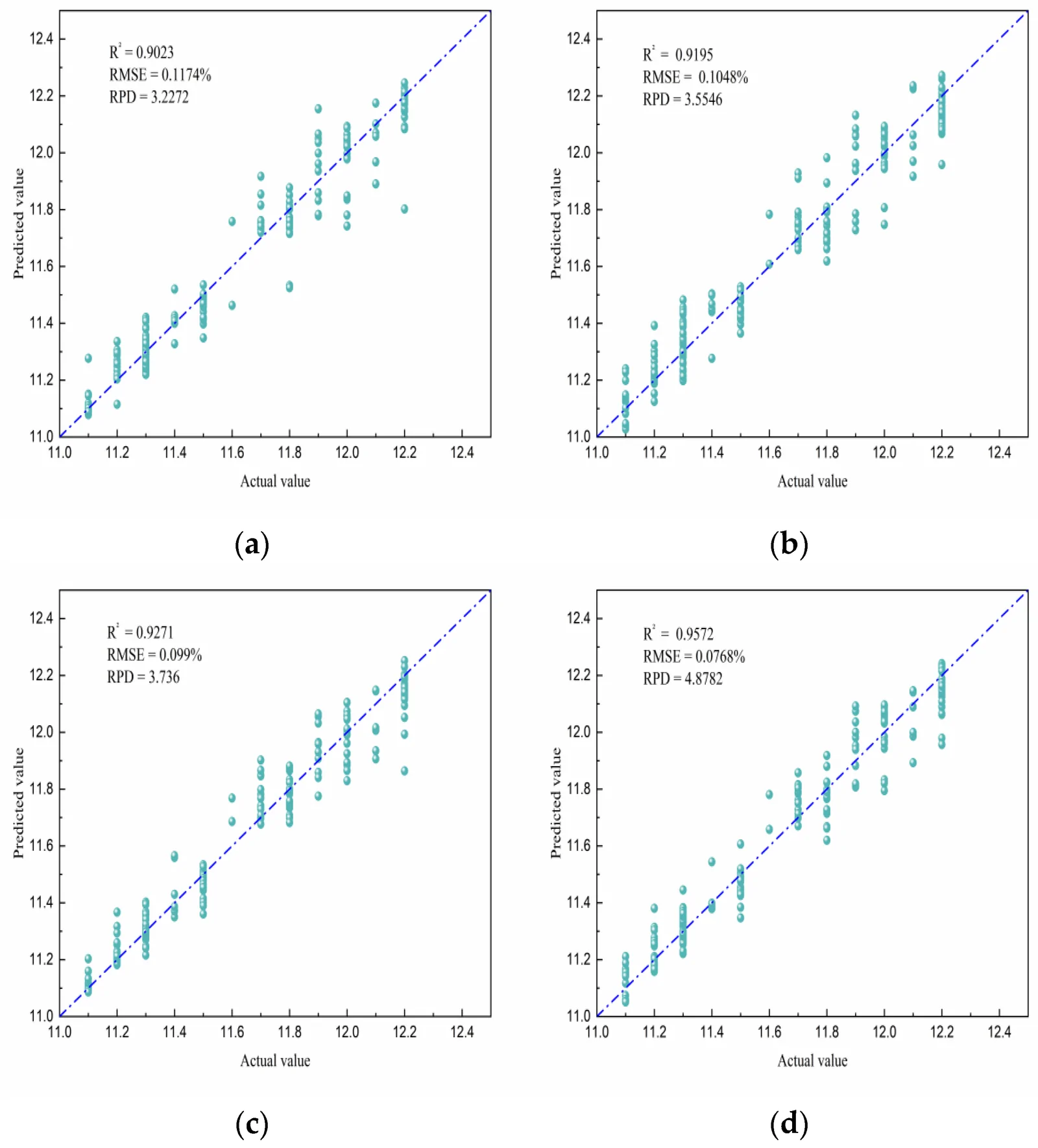
External validation of the optimal models: (**a**) GPR, (**b**) UVE-SPA-GPR, (**c**) SGD-GPR, and (**d**) SGD-UVE-SPA-GPR.

To further validate the robustness and generalization performance of the optimal SGD-UVE-SPA-GPR model, this study additionally collected 200 camel milk samples from five farms in April 2025 using a unified sampling protocol, which served as an independent external validation set. All data preprocessing, feature variable selection (UVE, SPA, and UVE-SPA), and model hyperparameter optimization were performed solely using the calibration set. The preprocessing parameters, optimal feature variables, and hyperparameters were fully fixed prior to external validation. As shown in Figure 9, the model achieved excellent generalization performance, with an *R*^2^ of 0.9572 and an RMSE of 0.0768%. These results confirm that the combination of SGD and UVE-SPA not only enhances the predictive accuracy of GPR but also improves its generalization capability, thereby validating the model’s applicability for predicting camel milk SNF. In Figure 9, the turquoise circles represent the sample points, and the blue dashed line represents the ideal fitting line where the predicted values exactly match the true values. All sample points are closely distributed along this diagonal, with no significant deviation, indicating that the models possess sound quantitative predictive capability for camel milk SNF. Without any preprocessing or feature variable extraction, the GPR model achieved an *R*^2^ of 0.9023, an RMSE of 0.1174%, and an RPD of 3.2272. After feature variable extraction using UVE-SPA, the model performance improved slightly, with *R*^2^ = 0.9195, RMSE = 0.1048%, and RPD = 3.5546. Following SGD preprocessing, the SGD-GPR model was further enhanced, yielding *R*^2^ = 0.9271, RMSE = 0.099%, and RPD = 3.7360. The SGD-UVE-SPA-GPR model was identified as the optimal model, achieving an *R*^2^ of 0.9572, an RMSE of 0.0768%, and an RPD of 4.8782. The RPD value of the optimal model exceeds 4, indicating excellent quantitative predictive capability. The sample points closely aligned with the ideal fitting line, with no significant systematic deviation observed across either low or high concentration ranges. In comparison with the other three models, the SGD-UVE-SPA-GPR model achieved the best overall performance on the external validation set by eliminating baseline noise through SGD spectral preprocessing and removing redundant dielectric spectral variables via UVE-SPA feature selection, thereby reducing interference from uninformative information and demonstrating robust generalization stability.

#### Residual Plot for the External Validation of the Optimal Model

**Figure 10:**
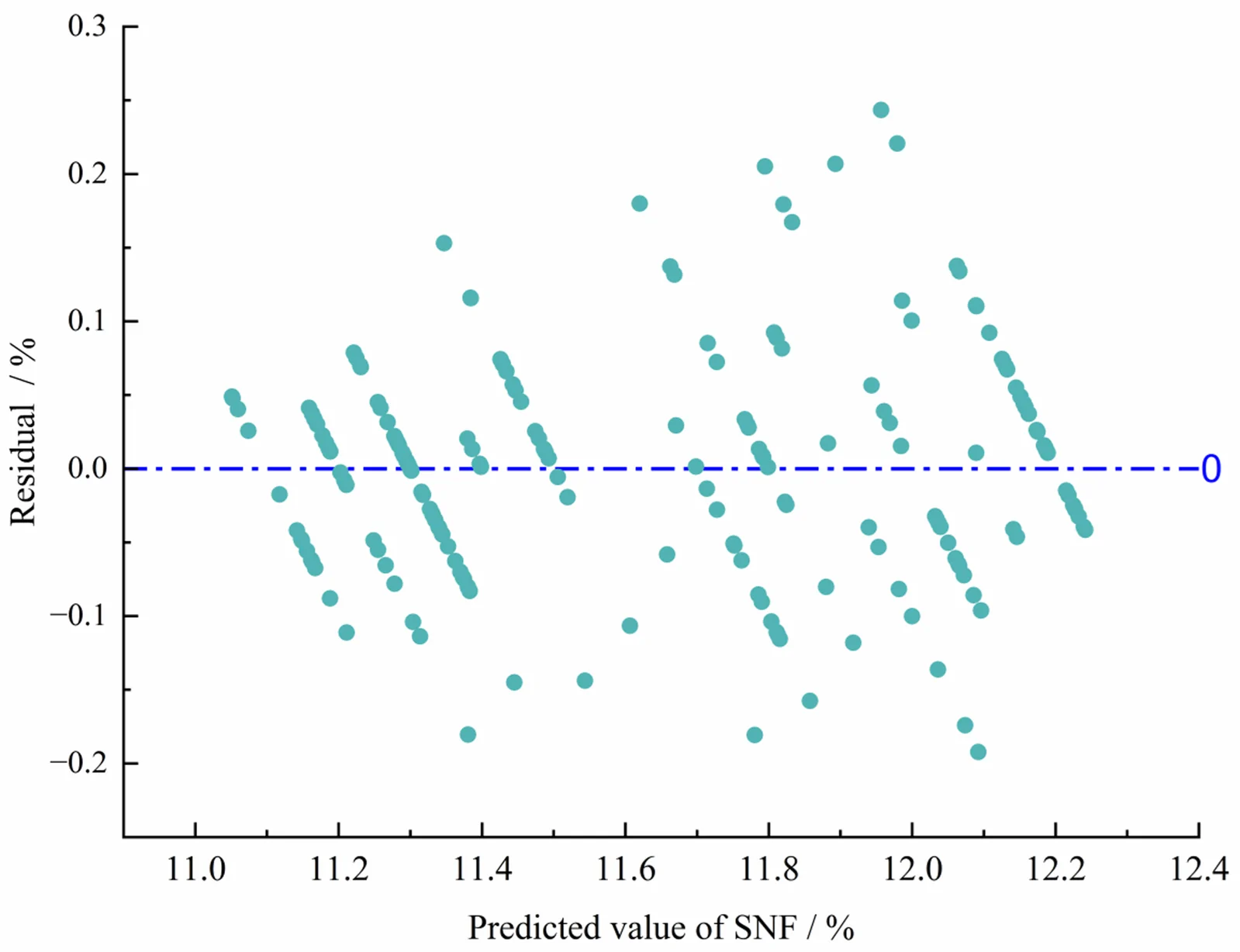
Residual-versus-predicted-value distribution for the optimal model on the external validation set.

To further evaluate the robustness and generalization ability of the SGD-UVE-SPA-GPR model, a residual-versus-predicted-value plot was constructed based on the 200 external validation samples, with the results presented in Figure 10. The residuals of the external validation samples were randomly distributed on both sides of the zero-residual baseline (blue dashed line), without a systematic tendency to cluster on one side, indicating that the model does not exhibit significant systematic prediction bias for the independent external samples. As the predicted camel milk SNF values increased, the residual fluctuation range did not expand appreciably, and no significant heteroscedasticity was observed. Although a small number of discrete sample points with relatively large absolute residual values were present, the vast majority of residuals fell within the range of −0.2% to 0.2%, indicating a generally low overall error level. These results collectively confirm that the model maintains sound predictive capability for new camel milk samples collected from different farms, demonstrating satisfactory generalization performance.

## 4. Discussion

In this study, the ε′ and ε″ of camel milk were collected at 201 frequency points over the frequency range of 0.1–26.5 GHz. By combining preprocessing with feature variable extraction methods, PLSR, SVM, LSTM, and GPR models were established for SNFC prediction. The prediction accuracies of the established models were compared, and SGD-UVE-SPA-GPR was identified as the optimal model for predicting camel milk SNFC. Yang et al. [52] utilized near-infrared spectroscopy combined with machine learning models to predict the SNFC of milk, achieving an $R_{P}^{2}$ of 0.92 and an RMSEP of 0.076; these prediction results are lower than those of the present study ($R_{P}^{2}$ = 0.9685, RMSEP = 0.0541%). This discrepancy may be attributed to the fact that near-infrared spectroscopy is susceptible to sample heterogeneity when detecting SNF in milk: the presence of fat globules and fat biofilms in the emulsion enhances light scattering effects and limits penetration depth, thereby weakening the correlation between the spectral signal and the concentration of internal components [53]. In contrast, dielectric spectroscopy is based on the principle of interaction between electromagnetic waves and molecular polarization as well as ionic conduction of materials. It is not affected by the heterogeneity of the milk system or scattering from fat globules, as electromagnetic waves can penetrate the entire milk sample. This technique can stably capture the low-frequency ionic conduction and high-frequency dipole relaxation features generated by components such as proteins, lactose, and mineral salts, thereby providing a more authentic representation of the sample’s internal compositional information [54]. Consequently, dielectric spectroscopy effectively overcomes the penetration depth limitations of spectroscopic methods in dairy product detection and offers a superior physical basis for quantitative prediction of camel milk SNFC, resulting in more accurate prediction performance. Liu et al. [55] investigated the variation patterns of ε′ and ε″ of milk samples with different SNF contents over the frequency range of 20–4500 MHz. However, that study was limited to qualitative analysis of dielectric properties and merely described superficial variations in dielectric parameters with compositional changes, without establishing a quantitative prediction model for SNFC. In contrast, the present study combines dielectric spectroscopy with chemometric methods to develop a high-precision quantitative prediction model for SNFC. The establishment of this model can provide key technical support for achieving rapid, non-destructive, and online detection of camel milk quality. Fang et al. [14] employed dielectric spectroscopy combined with a single feature variable extraction method to establish an RPS-SVR model for predicting the protein content of bovine milk, achieving an increase in $R_{P}^{2}$ of 0.10%, a decrease in RMSEP of 2.60%, and an increase in RPDP of 3.08%. In contrast, the present study utilizes dielectric spectroscopy combined with preprocessing and feature variable extraction methods for SNFC detection, yielding significantly higher prediction accuracy than that of Fang et al. with an $R_{P}^{2}$ increase of 6.96%, an RMSEP reduction of 39.82%, and an RPDP increase of 73.04%. It can be seen that a single feature selection method can only extract a limited amount of effective spectral information and is unable to filter out interfering signals such as baseline drift and high-frequency noise during dielectric measurements. In contrast, the present study employed SGD preprocessing combined with UVE-SPA feature variable extraction. Specifically, SGD was first applied to smooth the spectra, remove noise, and correct baseline shifts, thereby recovering the authentic dielectric response signals. Subsequently, UVE-SPA was used to precisely identify the core frequency variables that are highly correlated with the ionic relaxation and polarization characteristics related to SNFC, thereby eliminating redundant and irrelevant spectral information to the greatest extent and accurately matching the dielectric response patterns of camel milk solids. Therefore, compared with a single feature variable extraction method, the combined approach of preprocessing and feature variable extraction offers distinct advantages in terms of signal fidelity, feature focus, and anti-interference capability, ultimately achieving substantial improvements in both prediction accuracy and model stability.

This study proposes a quantitative detection method for camel milk SNFC that combines preprocessing with feature variable extraction. This method can serve as a methodological reference for non-destructive detection research on other dairy products and agricultural commodities. However, the current study still has some limitations. The samples used in this study were collected exclusively from Xinjiang, China, and the model has not yet been validated on camel milk samples from other production regions. Consequently, the model trained in this study is not directly transferable to other samples, which would require recalibration and model rebuilding. All experiments were conducted under uniform conditions, and the effects of factors such as sample temperature, lactation stage, animal feed, and storage conditions on the dielectric signals and model performance were not separately investigated. Therefore, the current model is only applicable to the detection of SNFC in camel milk from Xinjiang under conditions similar to those of this study. For future industrial application, the sample database should be expanded to include camel milk from different production regions and breeds. Meanwhile, targeted experiments should be designed to investigate the effects of the aforementioned environmental and husbandry-related factors, so as to further enhance the adaptability and applicability of the prediction model.

The dielectric spectroscopy method employed in this study requires no chemical reagents, involves simple sample preparation, and offers rapid detection, thus possessing significant potential for real-time on-site monitoring and quality control in both pasture and processing plant settings. It can therefore provide a theoretical basis and technical support for the development of automated detection equipment for camel milk quality assessment. Based on the effective detection frequency band and optimal modeling strategy identified in this study, future work will focus on developing portable automated detection equipment suitable for field and processing conditions by selecting low-cost radio-frequency components and simplifying the equipment calibration procedure, thereby further enhancing the practical applicability of dielectric spectroscopy technology.

## 5. Conclusions

This study proposes a quantitative detection method for camel milk SNFC based on dielectric spectroscopy combined with preprocessing and feature variable extraction. Compared with direct modeling using raw dielectric spectra, SNV, SGS, and SGD preprocessing can eliminate irrelevant interferences such as baseline drift and random noise, while UVE, SPA, and UVE-SPA feature variable extraction can remove redundant collinear variables and select dielectric features that are strongly correlated with SNFC. Consequently, the prediction accuracy of models was improved after the combination of preprocessing and feature variable extraction. Among all methods, SGD preprocessing can effectively correct baseline shifts and scattering interference in the dielectric signals, while UVE-SPA feature variable extraction ensures both feature effectiveness and feature set parsimony. Combined with the excellent nonlinear fitting capability of GPR, these advantages enabled the SGD-UVE-SPA-GPR model to achieve the best predictive performance, with $R_{P}^{2}$ = 0.9685, RMSEP = 0.0541%, and RPDP = 5.6780. In the future, we will continue to optimize the existing machine learning models and develop automated detection equipment suitable for practical deployment in dairy processing plants, so as to meet the demands of dairy enterprises and consumers for high-quality camel milk.

## Figures and Tables

**Table 1:** Statistical analysis of camel milk SNFC from different regions.

Sampling Area	Sample Size	Max	Min	Mean ± SD
Keping County, Aksu Prefecture	40	11.50	11.20	11.32 ± 0.09 c
7th Regiment, First Division	40	12.20	11.4	11.90 ± 0.33 a
Fuhai County, Altay Prefecture	40	11.90	11.10	11.50 ± 0.30 b
Huocheng County, Ili Prefecture	40	11.50	11.40	11.48 ± 0.04 b
Yiwu County, Hami City	40	11.50	11.20	11.33 ± 0.08 c
Total of All Samples	200	12.20	11.10	11.51 ± 0.29

Note: SNFC is expressed in percentage (%); SD is Standard Deviation. a: SNFC from the 7th Regiment of the First Division > other four regions *p* < 0.05. b: Fuhai and Huocheng: no significant difference between them; significantly < a, but > c. c: Keping and Yiwu: no significant difference between them; significantly < a and b.

**Table 2:** Prediction results of camel milk SNFC based on feature variable extraction methods.

Model	Feature Extraction Method	Number of Features	Calibration Set			Prediction Set		
			*R* ^2^	RMSE(%)	RPD	*R* ^2^	RMSE(%)	RPD
PLSR	FDS	402	0.9745	0.0469	6.2918	0.9017	0.0920	3.2168
	SPA	20	0.9321	0.0716	3.8528	0.9056	0.1024	3.2826
	UVE	35	0.9325	0.0745	3.8621	0.9022	0.0964	3.2247
	UVE-SPA	30	0.9316	0.0768	3.8392	0.9291	0.0781	3.7880
SVM	FDS	402	0.9936	0.0231	12.5727	0.8902	0.1006	3.0434
	SPA	20	0.9377	0.0732	4.0197	0.9018	0.0927	3.2179
	UVE	42	0.9268	0.0776	3.7084	0.9045	0.0958	3.2637
	UVE-SPA	30	0.9370	0.0739	4.0007	0.9114	0.0867	3.3885
LSTM	FDS	402	0.9316	0.0766	3.8379	0.9091	0.0886	3.3259
	SPA	20	0.9179	0.0839	3.5043	0.9135	0.0864	3.4091
	UVE	42	0.9212	0.0812	3.5752	0.9163	0.0850	3.4666
	UVE-SPA	30	0.9338	0.0754	3.9024	0.9207	0.0827	3.5601
GPR	FDS	402	0.9774	0.0441	6.6872	0.9055	0.0899	3.2813
	SPA	20	0.9417	0.0698	4.1598	0.9139	0.0891	3.4367
	UVE	42	0.9553	0.0620	4.7483	0.9196	0.0832	3.5574
	UVE-SPA	30	0.9602	0.0583	5.0307	0.9266	0.0804	3.7225

**Table 3:** Prediction results of camel milk SNF based on SNV combined with feature variable extraction methods.

Model	Feature Extraction Method	Number of Features	Calibration Set			Prediction Set		
			*R* ^2^	RMSE(%)	RPD	*R* ^2^	RMSE(%)	RPD
PLSR	FDS	402	0.9571	0.0624	4.8427	0.9044	0.0843	3.2606
	SPA	31	0.9459	0.0668	4.3135	0.9283	0.0821	3.7670
	UVE	55	0.9505	0.0676	4.5102	0.9217	0.0747	3.6037
	UVE-SPA	52	0.9448	0.0699	4.2714	0.9354	0.0726	3.9675
SVM	FDS	402	0.9962	0.0179	16.3614	0.9193	0.0845	3.5505
	SPA	31	0.9311	0.0765	3.8220	0.9228	0.0833	3.6296
	UVE	55	0.9638	0.0551	5.2751	0.9321	0.0788	3.8706
	UVE-SPA	25	0.9562	0.0614	4.7969	0.9527	0.0638	4.6413
LSTM	FDS	402	0.9114	0.0861	3.3729	0.9101	0.0880	3.3452
	SPA	20	0.9245	0.0795	3.6547	0.9205	0.0828	3.5565
	UVE	40	0.9305	0.0774	3.8084	0.9195	0.0833	3.5354
	UVE-SPA	25	0.9386	0.0724	4.0509	0.9345	0.0752	3.9187
GPR	FDS	402	0.9683	0.0523	5.6347	0.9105	0.0882	3.3700
	SPA	31	0.9524	0.0645	4.5996	0.9390	0.0717	4.0835
	UVE	55	0.9500	0.0645	4.4892	0.9374	0.0766	4.0303
	UVE-SPA	25	0.9528	0.0633	4.6169	0.9447	0.0705	4.2894

**Table 4:** Prediction results of camel milk SNF based on SGS combined with feature variable extraction methods.

Model	Feature Extraction Method	Number of Features	Calibration Set			Prediction Set		
			*R* ^2^	RMSE(%)	RPD	*R* ^2^	RMSE(%)	RPD
PLSR	FDS	396	0.9538	0.0604	4.6704	0.9008	0.1013	3.2020
	SPA	20	0.9365	0.0741	3.9809	0.9179	0.0842	3.5186
	UVE	71	0.9501	0.0650	4.4928	0.9263	0.0816	3.7138
	UVE-SPA	50	0.9416	0.0696	4.1521	0.9308	0.0808	3.8320
SVM	FDS	396	0.9725	0.0473	6.0532	0.9077	0.0951	3.3188
	SPA	20	0.9224	0.0819	3.6044	0.9104	0.0875	3.3706
	UVE	71	0.9328	0.0745	3.8724	0.9193	0.0874	3.5505
	UVE-SPA	60	0.9342	0.0752	3.9152	0.9294	0.0783	3.7955
LSTM	FDS	396	0.9379	0.0713	4.0292	0.9148	0.0858	3.4345
	SPA	30	0.9375	0.0730	4.0172	0.9248	0.0805	3.6567
	UVE	71	0.9325	0.0764	3.8644	0.9262	0.0798	3.6907
	UVE-SPA	60	0.9460	0.0671	4.3201	0.9374	0.0735	4.0087
GPR	FDS	396	0.9582	0.0601	4.9097	0.9178	0.0840	3.5170
	SPA	20	0.9431	0.0700	4.2058	0.9277	0.0795	3.7502
	UVE	71	0.9536	0.0630	4.6585	0.9251	0.0814	3.6835
	UVE-SPA	60	0.9477	0.0659	4.3890	0.9393	0.0755	4.0949

**Table 5:** Prediction results of camel milk SNF based on SGD combined with feature variable extraction methods.

Model	Feature Extraction Method	Number of Features	Calibration Set			Prediction Set		
			*R* ^2^	RMSE(%)	RPD	*R* ^2^	RMSE(%)	RPD
PLSR	FDS	396	0.9590	0.0598	4.9581	0.9114	0.0861	3.3884
	SPA	30	0.9359	0.0727	3.9631	0.9324	0.0784	3.8791
	UVE	150	0.9574	0.0590	4.8639	0.9392	0.0770	4.0895
	UVE-SPA	60	0.9553	0.0603	4.7513	0.9502	0.0696	4.5203
SVM	FDS	396	0.9977	0.0137	21.3586	0.9123	0.0880	3.4071
	SPA	20	0.9558	0.0620	4.7709	0.9420	0.0704	4.1862
	UVE	34	0.96073	0.0551	5.0646	0.9390	0.0727	4.0860
	UVE-SPA	20	0.9598	0.0572	5.0022	0.9598	0.0627	5.0306
LSTM	FDS	396	0.9231	0.0802	3.6199	0.9123	0.0870	3.3855
	SPA	20	0.9550	0.0607	4.7321	0.9458	0.0683	4.3099
	UVE	42	0.9560	0.0619	4.7855	0.9462	0.0681	4.3221
	UVE-SPA	25	0.9645	0.0543	5.3273	0.9585	0.0598	4.9246
GPR	FDS	396	0.9833	0.0380	7.7766	0.9290	0.0775	3.7867
	SPA	20	0.9780	0.0425	6.7650	0.9503	0.0691	4.5238
	UVE	42	0.9764	0.0449	6.5326	0.9505	0.0662	4.5304
	UVE-SPA	20	0.9781	0.0428	6.7790	0.9685	0.0541	5.6780

## Data Availability

The original contributions presented in the study are included in the article/Supplementary Material. Further inquiries can be directed to the corresponding author.

## Supplementary Materials

The following supporting information can be downloaded at: https://www.mdpi.com/article/10.3390/foods15203584/s1, Table S1: Table of Model-Selected Frequencie.

## Abbreviations

The following abbreviations are used in this manuscript:

SNFC	Solids-not-fat content
SNV	Standard normal variate
SGS	Savitzky–Golay smoothing
SGD	Savitzky–Golay derivative
UVE	Uninformative variables elimination
SPA	Successive projections algorithm
FDS	Full dielectric spectra
SNF	Solids-not-fat
PLSR	Partial least squares regression
SVM	Support vector machine
LSTM	Long short-term memory
GPR	Gaussian process regression
*R*^2^	Coefficient of Determination
RMSE	Root Mean Square Error
RPD	Residual Predictive Deviation
